# Subsidence of Cholera

**Published:** 1849-10

**Authors:** 


### SUBSIDENCE OF CHOLERA.

From all parts of the country we receive the gratifying intelligence of the decline of this destructive epidemic. Louisville, it is known to our readers, has experienced only the slightest visitation of the disease. For more than a month past, not a case has been reported in the city, and our citizens, at present, enjoy the most perfect exemption from complaints of every form and grade.

